# Increased mTORC2 pathway activation in lymph nodes of iMCD‐TAFRO

**DOI:** 10.1111/jcmm.17251

**Published:** 2022-04-30

**Authors:** Alexis D. Phillips, Joseph J. Kakkis, Patricia Y. Tsao, Sheila K. Pierson, David C. Fajgenbaum

**Affiliations:** ^1^ Center for Cytokine Storm Treatment & Laboratory Department of Medicine University of Pennsylvania Philadelphia Pennsylvania USA

**Keywords:** autoimmune lymphoproliferative syndrome, Castleman disease, idiopathic multicentric Castleman disease, iMCD, mTOR, mTORC2, pNDRG1, TAFRO

## Abstract

Idiopathic multicentric Castleman disease (iMCD) is a rare and life‐threatening haematologic disorder involving polyclonal lymphoproliferation and organ dysfunction due to excessive cytokine production, including interleukin‐6 (IL‐6). Clinical trial and real‐world data demonstrate that IL‐6 inhibition is effective in 34–50% of patients. mTOR, which functions through mTORC1 and mTORC2, is a recently discovered therapeutic target. The mTOR inhibitor sirolimus, which preferentially inhibits mTORC1, has led to sustained remission in a small cohort of anti‐IL‐6‐refractory iMCD patients with thrombocytopenia, anasarca, fever, renal dysfunction and organomegaly (iMCD‐TAFRO). However, sirolimus has not shown uniform effect, potentially due to its limited mTORC2 inhibition. To investigate mTORC2 activation in iMCD, we quantified the mTORC2 effector protein pNDRG1 by immunohistochemistry of lymph node tissue from six iMCD‐TAFRO and eight iMCD patients who do not meet TAFRO criteria (iMCD‐not‐otherwise‐specified; iMCD‐NOS). mTORC2 activation was increased in all regions of iMCD‐TAFRO lymph nodes and the interfollicular space of iMCD‐NOS compared with control tissue. Immunohistochemistry also revealed increased pNDRG1 expression in iMCD‐TAFRO germinal centres compared with autoimmune lymphoproliferative syndrome (ALPS), an mTOR‐driven, sirolimus‐responsive lymphoproliferative disorder, and comparable staining between iMCD‐NOS and ALPS. These results suggest increased mTORC2 activity in iMCD and that dual mTORC1/mTORC2 inhibitors may be a rational therapeutic approach.

## 1 INTRODUCTION

Idiopathic multicentric Castleman disease (iMCD) is a rare and heterogeneous lymphoproliferative disorder characterized by multifocal lymphadenopathy and cytokine‐driven systemic inflammation. Patients with the most severe form of iMCD present with thrombocytopenia, anasarca, fever, reticulin fibrosis and organomegaly (iMCD‐TAFRO).^1^ Other iMCD patients, referred to as iMCD‐not‐otherwise‐specified (iMCD‐NOS), often demonstrate thrombocytosis, hypergammaglobulinemia and a milder clinical course.^2^ Aberrant interleukin‐6 (IL‐6) signalling is a known disease driver in some cases and anti‐IL‐6 therapy is recommended first‐line for iMCD,^3^ but two‐thirds of patients did not respond to anti‐IL‐6 monoclonal antibody treatment with siltuximab in the phase II trial.^4^

The mammalian target of rapamycin (mTOR) pathway is a key regulator of cell proliferation, activation and survival and a recently discovered therapeutic target in iMCD. mTOR is a kinase that integrates inputs from various cytokines, growth factors and other ligands and signals through mTOR complex 1 (mTORC1) and mTOR complex 2 (mTORC2).^5^ Analysis of mTORC1 activity in iMCD, including immunohistochemistry (IHC) of the mTORC1 marker pS6 in iMCD lymph nodes, has revealed elevated activity of mTORC1 across multiple iMCD cohorts.^6^ The mTOR inhibitor sirolimus, which preferentially inhibits mTORC1 compared to mTORC2, has been associated with durable clinical remission in three published cases of anti‐IL‐6‐refractory iMCD‐TAFRO, and a clinical trial is underway to assess its efficacy in iMCD (NCT03933904).^7^ However, interrogation of data from the ACCELERATE registry found that only 5/11 (45.5%) of iMCD cases treated with sirolimus with or without other agents have achieved a response (unpublished data). This suggests that sirolimus is not uniformly effective in iMCD, potentially due to its limited ability to inhibit mTORC2, which has not been studied in iMCD. Autoimmune lymphoproliferative syndrome (ALPS) is an mTOR‐driven lymphoproliferative disorder that uniformly responds to sirolimus, suggesting involvement of mTORC1 in pathogenesis.^8^ Increased mTORC2 activation in iMCD relative to normal controls and ALPS would suggest a potential mechanism of resistance that could bypass mTORC1 suppression with sirolimus. Thus, further investigation into the role of mTORC2 in iMCD pathogenesis is warranted.

Though little is known about mTORC2 biology, it is thought to regulate cell survival, proliferation and cytoskeletal pathways. Upon activation by growth factors in a PI3K‐dependent manner,^5^ mTORC2 phosphorylates and activates Akt and SGK1,^5^ which subsequently phosphorylates the cytoplasmic protein N‐Myc Downstream Regulated 1 (NDRG1) to pNDRG1.^9^ Thus, pNDRG1 expression is used as a reliable biomarker of mTORC2 activation.^9^, ^10^, ^11^, ^12^, ^13^, ^14^ The involvement of mTORC2 in driving Akt‐mediated cell survival and anti‐apoptotic signalling in malignancies suggests a potential role of mTORC2 in lymphoproliferative disorders like iMCD.^15^, ^16^ Several dual mTORC1/2 inhibitors exist and could be considered if increased mTORC2 activation is found in iMCD.^15^ Herein, we quantify mTORC2 pathway activation in iMCD.

## 2 METHODS

### 2.1 Patient samples

Diagnostic lymph node samples were obtained from iMCD patients enrolled in the ACCELERATE Natural History Registry (NCT02817997).^17^ All subjects gave written informed consent, and the studies were approved by local institutional review boards in accordance with the provision of the Declaration of Helsinki and Good Clinical Practice Guidelines. Tissue from eight iMCD‐NOS and eight iMCD‐TAFRO patients was procured following formalin‐fixation paraffin embedding (FFPE).^18^, ^19^ Comprehensive clinical data and haematoxylin‐eosin stained lymph node slides were collected and reviewed by a panel of clinicians and pathologists for confirmation of an iMCD‐consistent diagnosis. Clinical and pre‐biopsy treatment data are reported in Table 1.

**TABLE 1:** Clinical characteristics of the iMCD cohorts

	iMCD‐TAFRO (N = 6)	iMCD‐NOS (N = 8)
Age, y; mean (range)	27 (1‐61)	38 (14‐58)
Sex, female:male	1:5	4:4
TAFRO criteria, present/assessed (%)		
Thrombocytopenia	6/6 (100)	2/4 (50)
Anasarca or oedema	6/6 (100)	1/7 (14)
Constitutional symptoms	6/6 (100)	6/8 (75)
Reticulin myelofibrosis	3/4 (75)	1/1 (100)
Renal dysfunction	4/6 (67)	0/5 (0)
Hepatomegaly and/or splenomegaly	4/6 (67)	3/7 (43)
Inflammation and organ dysfunction		
CRP, mg/dL	n = 5	n = 3
Mean (SD)	15.9 (9.9)	5.8 (7.6)
IL‐6, pg/mL	n = 5	No data
Mean (SD)	307.7 (92.3)	
VEGF, pg/mL	n = 3	No data
Mean (SD)	380.7 (107.0)	
IgG, mg/dL	n = 5	n = 2
Mean (SD)	1518 (951)	1525 (218)
Albumin, g/dL	n = 5	n = 4
Mean (SD)	2.5 (0.54)	3.6 (0.73)
Creatinine, mg/dL	n = 6	n = 4
Mean (SD)	1.2 (0.94)	1.0 (0.27)
Treatment prior to lymph node biopsy		
Intravenous immunoglobulin	2/6 (33)	0/8 (0)
Corticosteroids	1/6 (17)	1/8 (13)
Other treatments	0/6 (0)	0/8 (0)

Note

Each laboratory value represents the closest laboratory value to the date of diagnosis within 30 days prior to or following biopsy.

Abbreviations: CRP, C‐reactive protein; IgG, immunoglobulin G.; IL‐6, interleukin‐6; iMCD‐NOS, iMCD‐not‐otherwise‐specified; iMCD‐TAFRO, iMCD‐thrombocytopenia‐anasarca‐fever‐renal dysfunction‐organomegaly; SD, standard deviation; VEGF, vascular endothelial growth factor.

Eight metastasis‐free lymph nodes resected from breast cancer patients were selected as normal controls. Tissue samples from eight Hodgkin lymphoma‐not otherwise specified (HL‐NOS) and six ALPS lymph node samples were selected as a positive staining control and comparator group respectively. HL was selected because it has been shown to demonstrate increased mTORC2 expression.^20^ Tissue samples from all three comparator groups were procured from the University of Pennsylvania pathology department.

### 2.2 Immunohistochemistry (IHC)

IHC staining of 5‐μm‐thick formalin‐fixed paraffin‐embedded lymph node tissue samples was performed at the Pathology Core of the Children's Hospital of Philadelphia on a Leica Bond Max automated staining system (Leica Biosystems). Monoclonal rabbit anti‐pNDRG1 (Thr346) (Clone D98G11, Cell Signaling Technology, #5482) was used at a 1:100 dilution, according to the manufacturer's staining protocol. Haematoxylin counterstain was used to assess cell and tissue morphology.

### 2.3 pNDRG1 staining quantification

Image Analysis Toolkit software (colour deconvolution v9 algorithm) was used to estimate the staining strength of pixels for pNDRG1 (Figure S1). The percentage of areas with overall positive staining and weak, medium and strong staining was retrieved for each lymph node region (interfollicular space, germinal centres and mantle zones). The entire cross‐sectional area of each lymph node sample was analysed for pNDRG1 staining. One normal and two iMCD‐TAFRO samples were excluded from analysis due to non‐specific tissue artefact and insufficient tissue; 6 iMCD‐TAFRO, 8 iMCD‐NOS, 7 normal and 8 HL samples were included in the final analysis. For four HL‐NOS and one ALPS samples, only the interfollicular space was quantified due to lack of secondary follicles. The regions of each secondary follicle, including the germinal centre and mantle zone, and interfollicular space, were annotated and independently audited using Aperio ImageScope.

### 2.4 Statistical analysis

Percent positive staining was log2 transformed. For each staining region, equality of variance was tested, and T‐test with Bonferroni correction for multiple testing was performed. Analysis was performed using R 3.6.1.

## 3 RESULTS

**FIGURE 1:**
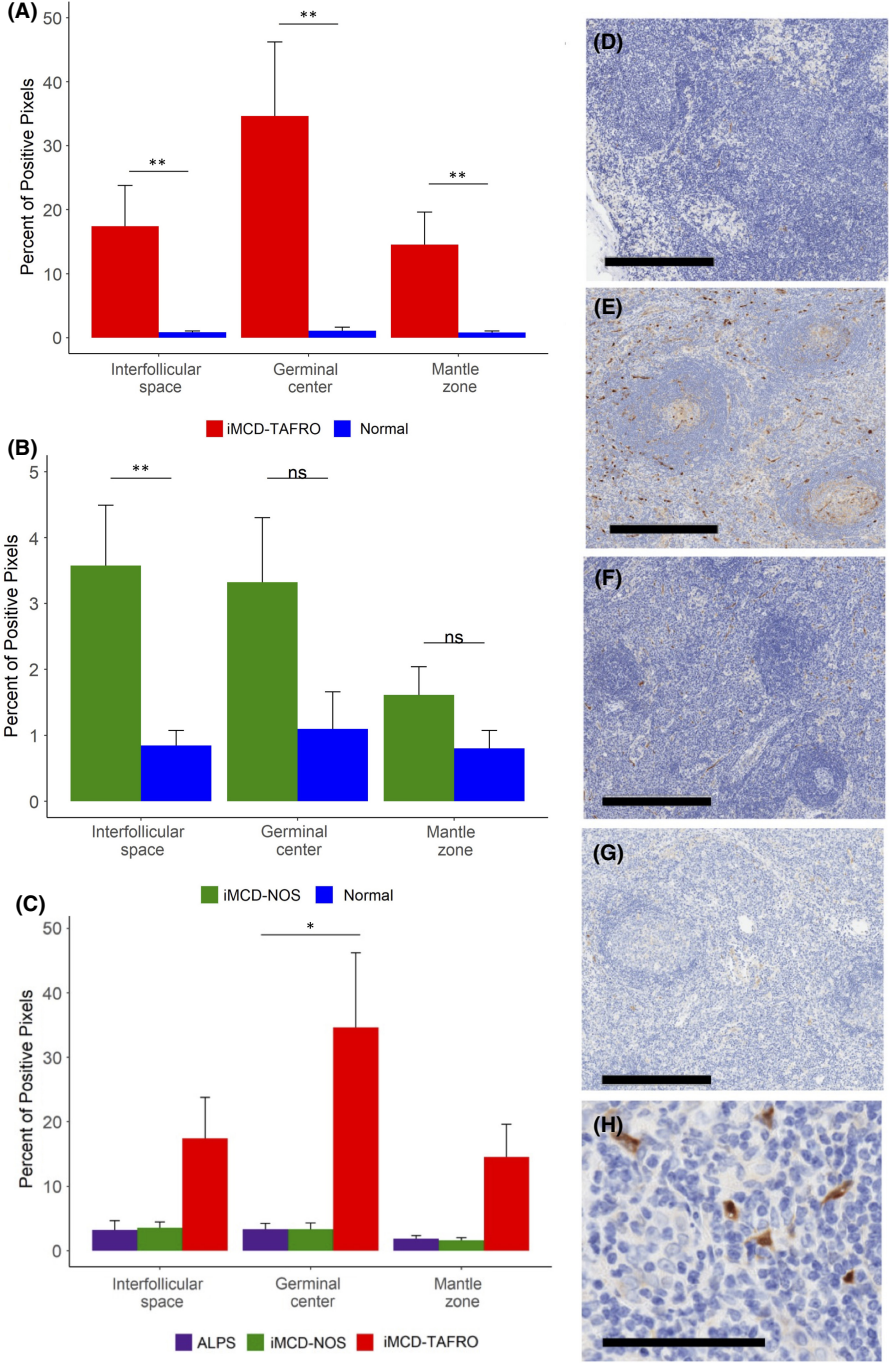
Comparison of pNDRG1 staining across iMCD subtypes and autoimmune lymphoproliferative syndrome (ALPS). (A) Stained pNDRG1 for iMCD‐TAFRO patients (n = 6) and (B) iMCD‐not otherwise specified (iMCD‐NOS) patients (n = 8) compared with a control group of metastasis‐free lymph nodes (normal) (n = 7). Relative to normal lymph nodes, pNDRG1 positive area was significantly elevated in the interfollicular space (*p* = 0.005), germinal centre (*p* = 0.002) and mantle zone (*p* = 0.007) of iMCD‐TAFRO lymph nodes. pNDRG1 positive area was significantly elevated in the interfollicular space (*p* = 0.005) of iMCD‐NOS lymph nodes relative to normal lymph nodes, but there was no difference in staining in the germinal centres (*p* = 0.59) and mantle zones (*p* = 0.30). (C) Stained pNDRG1 area for iMCD‐TAFRO patients (n = 6) and iMCD‐NOS patients (n = 8) compared with ALPS lymph nodes (n = 8). There was a significant increase in positive pNDRG1 staining in iMCD‐TAFRO germinal centres relative to ALPS (*p* = 0.02), and no difference in the interfollicular space (*p* = 0.18) and mantle zone (*p* = 0.11). There was no difference in positive pNDRG1 staining in iMCD‐NOS relative to ALPS in the interfollicular space (*p* = 1.0), germinal centres (*p* = 1.0) and mantle zones (*p* = 1.0). *p <.05, **p <.01. D‐G, Representative images of pNDRG1 (brown) staining for a (D) normal lymph node (E) iMCD‐TAFRO (F) iMCD‐NOS and (G) ALPS lymph node. Haematoxylin counterstain provides a blue nuclear stain to assess cell and tissue morphology. Bar = 400 μm. (H) The strongest pNDRG1‐positive cells appear to have spindle‐shaped morphology resembling stromal cells. Bar = 100 μm.

To investigate the degree of mTORC2 pathway activation in iMCD, we quantified pNDRG1 expression in iMCD‐TAFRO (N = 6), iMCD‐NOS (N = 8) and relevant controls. First, to confirm the assay's technical performance, we compared positive pNDRG1 staining in HL‐NOS (N = 8) relative to normal lymph nodes (N = 7) and found significantly elevated staining in interfollicular space (*p* = 0.049), germinal centre (*p* = 0.014) and mantle zone (*p* = 0.027) (Figure S2). Given that the assay demonstrated increased staining in the positive controls as expected, we sought to evaluate mTORC2 activation in iMCD‐TAFRO and iMCD‐NOS. Comparisons were performed separately due to the considerably different clinical phenotypes between iMCD‐TAFRO and iMCD‐NOS and our previous investigations of mTOR activity and sirolimus responsiveness in iMCD‐TAFRO.^6^ In iMCD‐TAFRO, pNDRG1 expression was significantly elevated in the interfollicular space (*p* = 0.005), germinal centres (*p* = 0.002) and mantle zones (*p* = 0.007) relative to normal lymph nodes (Figure 1A). Of note, we observed a high degree of variability in staining intensity among the iMCD‐TAFRO cases (Figure S3). Positive pNDRG1 staining was significantly increased in the interfollicular space (*p* = 0.005) of iMCD‐NOS lymph nodes relative to normal lymph nodes, but there was no difference in the germinal centres (*p* = 0.59) and the mantle zones (*p* = 0.30) (Figure 1B).

Next, we compared pNDRG1 expression in iMCD‐TAFRO and iMCD‐NOS to ALPS, a disease characterized by aberrant mTOR activation. While patients with ALPS consistently respond to sirolimus,^7^ anecdotal reports suggest that sirolimus is not uniformly effective in iMCD. We therefore hypothesized that mTORC2 signaling may be more highly elevated in iMCD, suggesting a potential mechanism of resistance that could circumvent mTORC1 inhibition. Our results revealed significantly increased pNDRG1 staining in iMCD‐TAFRO germinal centres relative to ALPS (*p* = 0.02) and non‐significantly increased staining in the interfollicular space (*p* = 0.18) and mantle zones (*p* = 0.11) (Figure 1C). There were no differences in pNDRG1 expression between iMCD‐NOS and ALPS in any region (Figure 1C). Representative images are shown in Figure 1D‐G.

We also made qualitative observations of cell morphology in iMCD lymph node samples stained for pNDRG1. We observed that many of the strongly positive pNDRG1 cells had spindle‐shaped morphology resembling stromal cells (Figure 1H).

Unfortunately, we could not assess associations between mTORC1 and mTORC2 activation due to limited sample size (only 8 of 14 iMCD patients in this study also had pS6 performed; Figure S4), as well as between mTORC2 activation and response to sirolimus in this cohort as only 1 patient received sirolimus.

## 4 DISCUSSION

In this study, we report the investigation of mTORC2 expression in iMCD. Our results demonstrate increased mTORC2 activity in iMCD‐TAFRO and iMCD‐NOS relative to normal lymph nodes, as well as increased mTORC2 activity in iMCD‐TAFRO relative to ALPS. There are a few potential implications of the increased mTORC2 activity observed in this study. First, mTORC2 may be a driver of iMCD pathogenesis. Given that sirolimus is a less potent inhibitor of mTORC2 relative to mTORC1, patients may benefit from dual mTORC1/mTORC2 or Janus kinase inhibition upstream of mTOR.^21^, ^22^ Alternatively, mTORC2 may have an additive effect that does not directly drive pathophysiology but instead contributes to worsening disease primarily due to another pathogenic driver. We previously found that sirolimus induced clinical benefit in 3 IL‐6 inhibitor‐refractory iMCD‐TAFRO patients, suggesting that, if present, mTORC2 elevation was not the primary driver in these patients.^7^ Lastly, mTORC2 activation may be unrelated to iMCD pathogenesis and activated as a bystander.

Notably, many of the strongly positive pNDRG1 cells had spindle‐shaped morphology resembling stromal cells. This result contrasts with the pS6‐positive cells in iMCD lymph nodes that have been shown to represent monocytes, plasma cells and as‐yet‐undefined cells with myeloid‐appearing morphology; a very small proportion of pS6‐positive cells in lymph node tissue appear to be T cells despite increased T‐cell activation in circulation.^6^ The likely difference in cell types with pS6‐ and pNDRG1‐staining suggests that mTORC1 and mTORC2 may be differentially active in different cell types. Our finding that the strongly positive pNDRG1 cells resemble stromal cells is also notable because stromal cell proliferation, particularly proliferation of follicular dendritic cells, is a common feature in iMCD lymph nodes.^23^ Further, we have previously identified elevated circulating levels of chemokines produced by stromal cells, including C‐X‐C motif chemokine ligand (CXCL) 13, C‐C motif chemokine ligand (CCL) 19 and CCL21 in iMCD flare compared to remission.^24^ Interestingly, these chemokines are primarily produced by two different types of stromal cells in different regions of the lymph node. CXCL13 is primarily produced by follicular dendritic cells located in the germinal centres and CCL19 and CCL21 are produced primarily by fibroblastic reticular cells in the interfollicular space. Though further studies are needed to uncover the specific cell types showing increased mTORC2 expression, the results support the involvement of stromal cells in mTORC2 activation and potentially iMCD pathogenesis.

Though we did not compare iMCD‐TAFRO and iMCD‐NOS directly, our results suggest that mTORC2 activation may differ between the two. This result should be confirmed and validated, especially given our previous finding demonstrating similar mTORC1 activation between both clinical subtypes of iMCD.^6^ Given the small sample size and degree of variability in this study, some comparisons are inconclusive and larger studies are needed. The relative paucity of information on mTORC2 biology complicates the understanding of these results in the context of iMCD.

Herein, we have demonstrated increased mTORC2 in iMCD‐TAFRO compared to normal lymph nodes and ALPS lymph nodes. Our results suggest that direct comparisons of iMCD‐TAFRO and iMCD‐NOS should be performed in a larger follow‐up study. Further studies are needed to confirm this finding, uncover cell types showing increased mTORC2 activity, and investigate therapeutic approaches.

## CONFLICTS OF INTEREST

D.C.F. has received research funding for the ACCELERATE registry (NCT02817997) from EUSA Pharma and consulting fees from EUSA Pharma, and Pfizer provides study drug with no associated research funding for the clinical trial of sirolimus (NCT03933904). D.C.F. has two provisional patents pending related to the diagnosis and treatment of iMCD. The remaining authors declare no competing interests.

## AUTHOR CONTRIBUTIONS

**Alexis Phillips:** Investigation (lead); Project administration (supporting); Visualization (equal); Writing – original draft (lead); Writing – review & editing (equal). **Joseph J Kakkis:** Conceptualization (supporting); Data curation (equal); Investigation (equal); Writing – original draft (lead); Writing – review & editing (equal). **Patricia Y Tsao:** Conceptualization (supporting); Methodology (lead); Project administration (lead); Resources (lead); Software (lead); Writing – review & editing (supporting). **Sheila Pierson:** Formal analysis (lead); Visualization (lead); Writing – review & editing (equal). **David C Fajgenbaum:** Conceptualization (lead); Formal analysis (equal); Investigation (equal); Methodology (lead); Writing – review & editing (equal).

## Supporting information

Supplementary Material

## Data Availability

The data that support the findings of this study are available from the corresponding author upon reasonable request.
