# Supplementary material for: Increased mTORC2 pathway activation in lymph nodes of iMCD‐TAFRO
Source: J Cell Mol Med. 2022 Apr 30;26(11):3147–52. doi: 10.1111/jcmm.17251 (PMC9170805; doi:10.1111/jcmm.17251)
Supplement: Supplementary file 1 — Supplementary Material [file JCMM-26-3147-s001.pdf]

Supplemental Data

Supplemental Figure 1.

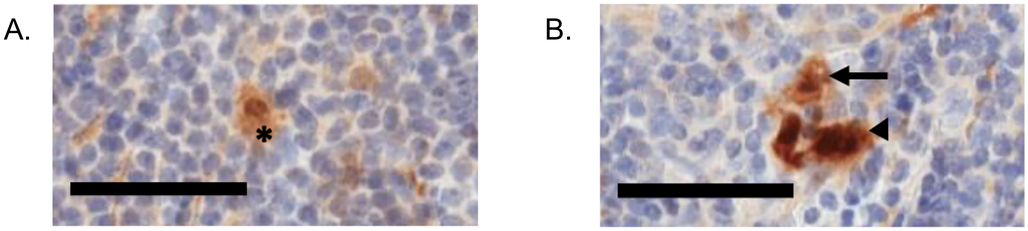

Supplemental Figure 1: **Cells used to establish thresholds for pNDRG1 staining strength.** Representative cells were selected to establish thresholds for **(A)** weak and **(B)** medium and strong staining of pixels for pNDRG1. Asterisk overlies the cell used to set the threshold for weak staining. Arrow and arrowhead point to the cells used to set the thresholds for medium and strong staining, respectively. Bar = 50 μm.

Supplemental Figure 2.

A.

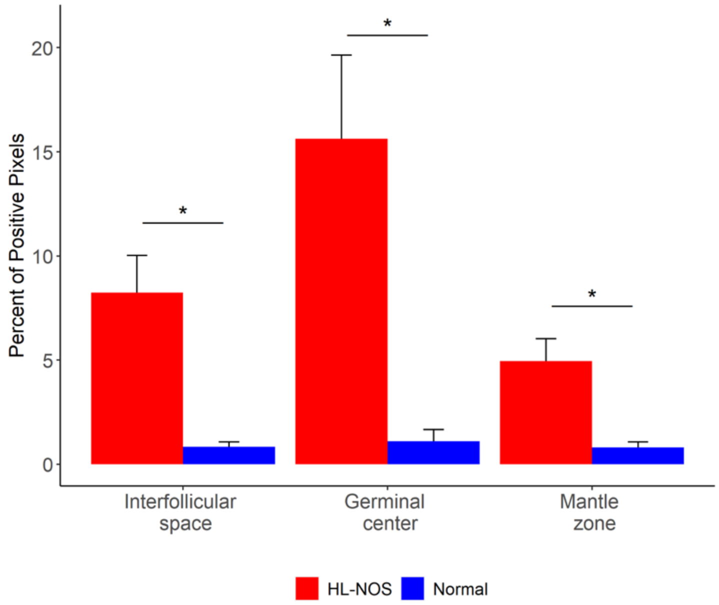

B.

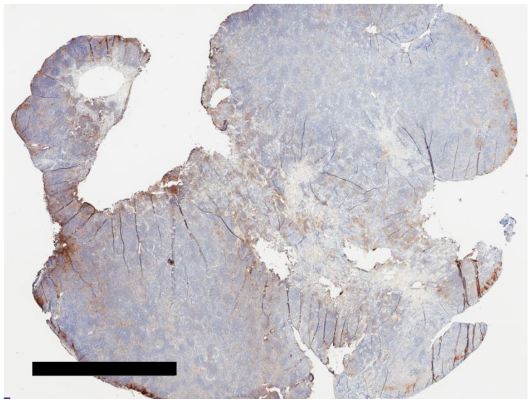

C.

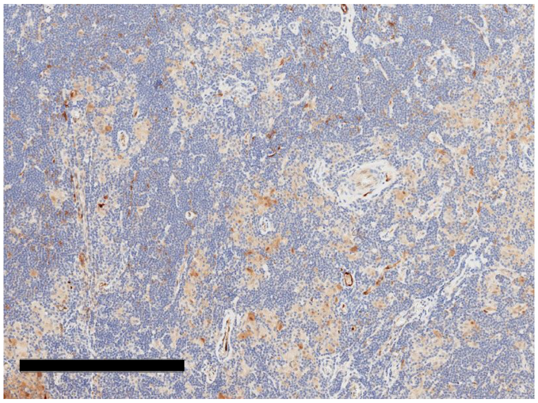

Supplemental Figure 2: **Comparison of pNDRG1 staining in Hodgkin lymphoma-not otherwise specified.** **(A)** Stained pNDRG1 for Hodgkin lymphoma-not otherwise specified (HL-NOS) patients (n = 8) compared with a control group of metastasis-free lymph nodes (normal lymph nodes) (n = 7). Relative to normal lymph nodes, pNDRG1 positive area was significantly elevated in the interfollicular space (P = 0.049), germinal center (P = 0.014), and mantle zone (P = 0.027) of HL-NOS lymph nodes. **(B-C)** Representative images of pNDRG1 (brown) staining for a HL-NOS lymph node. Hematoxylin counterstain provides a blue nuclear stain to assess cell and tissue morphology. (B) Low power view. Bar = 6 mm. (C) High power view. Bar = 400 µm.

Supplemental Figure 3.

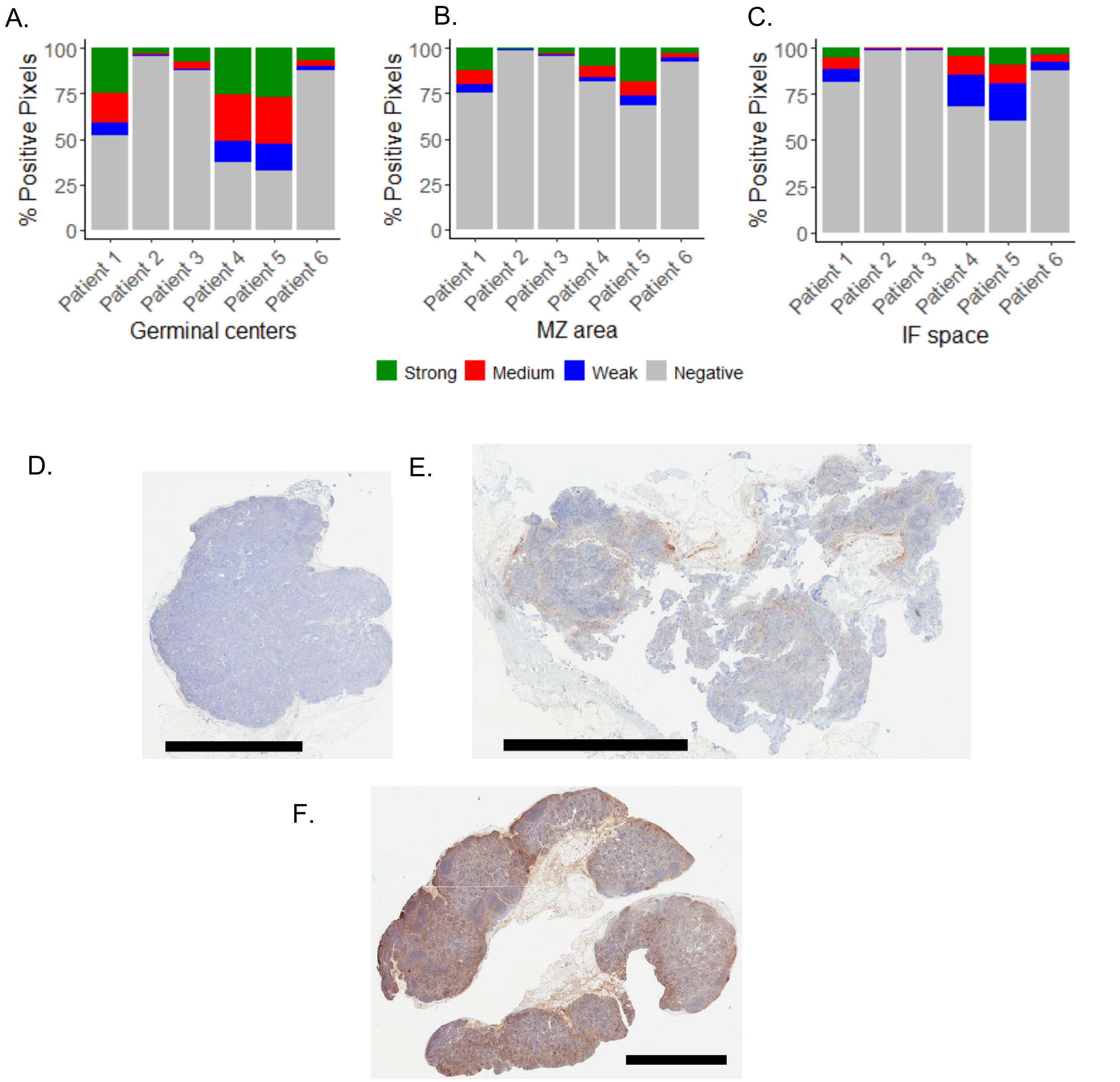

Supplemental Figure 3: **Variability of positive pNDRG1 staining within the iMCD-TAFRO cohort.** Percent positive weak, medium, and strong staining is demonstrated by patient for **(A)** germinal centers, **(B)** mantle zone area, and **(C)** interfollicular space. MZ=mantle zone, IF space=interfollicular space. Representative images of pNDRG1 (brown) staining is shown for iMCD-TAFRO lymph nodes with **(D)** weak (Patient 2) **(E)** intermediate (Patient 6) and **(F)** strong (Patient 5) levels of overall pNDRG1 staining. Hematoxylin counterstain provides a blue nuclear stain to assess cell and tissue morphology. Bar = 6 mm.

Supplemental Figure 4.

A.
B.
C.

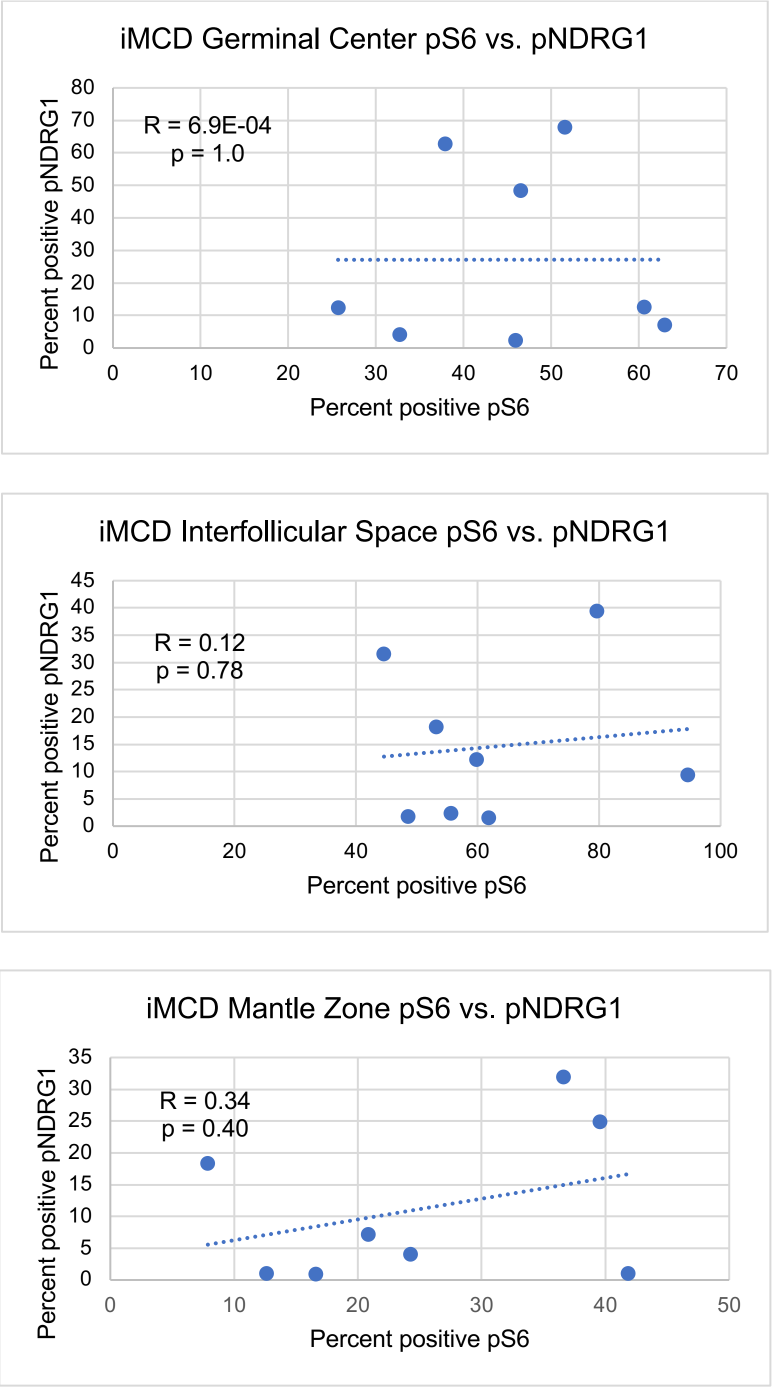

Supplemental Figure 4: **Plots for percent positive pNDRG1 staining versus percent positive pS6 staining in iMCD patients.** Scatter plots demonstrating the relationship between percent positive pNDRG1 staining and percent positive pS6 staining in the **(A)** germinal centers **(B)** interfollicular space and **(C)** mantle zones of 8 iMCD lymph node samples are shown. Each point represents one lymph node sample.
